# A Social Network Analysis of a Multi-sector Service System for Intimate Partner Violence in a Large US City

**DOI:** 10.1007/s10935-024-00774-2

**Published:** 2024-03-03

**Authors:** Xi Wang, Meredith Matone, Stephanie M. Garcia, Katherine S. Kellom, Deanna Marshall, Azucena Ugarte, Marcella Nyachogo, Samia Bristow, Peter F. Cronholm

**Affiliations:** 1 PolicyLab, Children’s Hospital of Philadelphia, 2716 South Street, 10-121, Philadelphia, PA 19146 USA; 2 Perelman School of Medicine, University of Pennsylvania, Philadelphia, PA USA; 3 Office of Domestic Violence Strategies of the City of Philadelphia, Philadelphia, PA USA; 4 Lutheran Settlement House, Philadelphia, PA USA; 5 Maternity Care Coalition, Philadelphia, PA USA; 6 Department of Family Medicine and Community Health, University of Pennsylvania, Philadelphia, PA USA; 7 Center for Public Health Initiatives, University of Pennsylvania, Philadelphia, PA USA; 8 Leonard Davis Institute of Health Economics, University of Pennsylvania, Philadelphia, PA USA

**Keywords:** Intimate partner violence, Social network analysis, Maternal and early childhood home visiting programs, Systems approach, Community-based agency

## Abstract

About one in four women in the US report having experienced some form of intimate partner violence (IPV) during their lifetime and an estimated 15.5 million children live in families in which IPV occurred in the past year. Families of young children with IPV experiences often face complex needs and require well-coordinated efforts among service providers across social and health sectors. One promising partnership aims to support pregnant and parenting IPV survivors through coordination between IPV agencies and community-based maternal and early childhood home visiting programs. This study used social network analysis (SNA) to understand the interconnectedness of the system of IPV prevention and intervention for families with young children in a large US city. The SNA included 43 agencies serving this population across various service domains spanning IPV, legal, maternal and child health, and public benefit programs. An SNA survey collected data on four forms of collaboration between agencies, including formal administrative relationship, referral reciprocity, case consultation, and shared activities in community committees/organizing bodies. Density and centrality were the primary outcomes of interest. A community detection analysis was performed as a secondary analysis. The overall level of interconnectedness between the 43 responding agencies was low. Making referrals to each other was the most common form of collaboration, with a network density of 30%. IPV agencies had the highest average number of connections in the networks. There was a high level of variation in external collaborations among home visiting agencies, with several home visiting agencies having very few connections in the community but one home visiting program endorsing collaborative relationships with upwards of 38 partner agencies in the network. In serving families at risk for IPV, home visiting agencies were most likely to have referral relationships with mental health provider agencies and substance use disorder service agencies. A community detection analysis identified distinct communities within the network and demonstrated that certain agency types were more connected to one another while others were typically siloed within the network. Notably, the IPV and home visiting communities infrequently overlapped. Sensitivity analyses showed that survey participants’ knowledge of their agencies’ external collaborations varied by their work roles and agencies overall had low levels of consensus about their connectedness to one another. We identified a heterogeneous service system available to families of young children at-risk for or experiencing IPV. Overall inter-agency connectedness was low, with many siloed agencies and a lack of shared knowledge of community resources. Understanding current collaborations, silos, and centrality of agencies is an effective public health tool for allocating scarce resources across diverse service sectors to efficiently improve the system serving families experiencing IPV.

## Background

While broad, estimates of US children who have witnessed intimate partner violence (IPV) in the home represent sizable exposure, ranging from 3.7 to 15.5 million, or 5 to 20 percent of all children (McDonald et al., 2006). The health and consequences of IPV are substantial: surviving IPV has been associated with both physical injury and psychological difficulties such as depression, posttraumatic stress disorder (PTSD) and substance abuse (Black, 2011; Breiding et al., 2014; Nathanson et al., 2012; Warshaw & Brashler, 2009). Children living in households characterized by frequent and severe IPV are at increased risk for psychological, social, emotional, and behavioral problems including mood and anxiety disorders, PTSD, substance use disorder (SUD), and school-related problems (Levendosky et al., 2013; Osofsky, 2003; Wathen & MacMillan, 2013). Intergenerational impacts additionally put children who witness IPV at higher risk of experiencing patterns of abuse in adulthood (Office on Women’s Health, https://www.womenshealth.gov/relationships-and-safety/domestic-violence/effects-domestic-violence-children).

Despite the magnitude of its influence, community-level public health responses to IPV, especially for families with young children, have been limited by resource deficits, interagency communication deficits, and siloed programming and outreach. As with other public health threats rooted in social factors, effective primary and secondary prevention strategies require well-coordinated efforts across social service and health sectors. In the 2006 American Journal of Public Health special issue on systems thinking, Leischow & Milstein described systems approaches as “a paradigm or perspective that considers connections among different components, plans for the implications of their interaction, and requires transdisciplinary thinking as well as active engagement of those who have a stake in the outcome to govern the course of change” (Leischow & Milstein, 2006). So-called “systems approaches” have gained momentum in areas within public health and their application to specific at-risk populations has included patients experiencing mental health issues (Becker et al., 1998), HIV (Kwait et al., 2001), and older age (Kaluzny et al., 1998).

While there is great need for maternal and child public health programs and social services to collaborate on a comprehensive systems approach to complex social determinants of health like IPV, cross-systems models of care are difficult to achieve due to fragmentation of services and limited resources to support innovation within the underfunded system in which these supports often function (Shorey et al., 2014; Sullivan, 2007). Improved programmatic partnerships between agencies would allow resources and expertise to be shared to facilitate efficient, effective, and comprehensive provision of services (Harris et al., 2008; Provan et al., 2005). Limited research to date has addressed systems-approaches to remediating proximate causes of maternal morbidity and mortality, including violence (Crear-Perry et al., 2021; Wang et al., 2020). To improve cross-systems alignment for families experiencing IPV, we must first understand how existing systems operate across the various sectors involved in prevention and intervention of family violence, including public health, social service, legal, and immigration sectors.

One promising partnership aims to support pregnant and parenting IPV survivors through coordination between IPV agencies and maternal and early childhood home visiting programs. Evidence-based home visiting programs are a public health intervention providing voluntary in-home services and support for parents and their children from birth to age five. These services are present in 1054 US counties, serving approximately 140,000 parents and children in 2021 (Maternal and Child Health Bureau, https://mchb.hrsa.gov/maternal-child-health-initiatives/home-visiting-overview). Because of the increased risk of IPV faced by pregnant women and new mothers, home visits offer an opportunity to deliver services designed to improve IPV outcomes within a trusting relationship with the home visitor (National Home Visiting Resource Center, 2021; Niland et al., 2020). Home visitors can support families through empowerment strategies including supporting the identification of signs of IPV during home visits, increasing awareness among IPV with survivors who may not recognize aspects of abusive relationships, and connecting often isolated survivors to services and resources (Dauber et al., 2017; Goldberg et al., 2018). When home visiting agencies do prioritize special populations of families, including those experiencing IPV, they can have improved systems for service coordination and referral activities (West et al., 2021). Home visiting programs, if adequately connected with local IPV agencies and ancillary supports, could provide pathways to creating a systems approach to preventing and addressing IPV among expectant families and those with young children.

The scope and interconnectivity of the service network available and accessible to families experiencing IPV engaged in home visiting remains unclear. The goal of this project is to understand and describe the landscape of IPV prevention and intervention in Philadelphia, Pennsylvania for families with young children by examining the connections between local agencies servicing families experiencing or at risk for IPV in order to inform the development of viable systems interventions. We used social network analysis (SNA), a method uniquely suited to examining connections (Borgatti et al., 2009). For decades, the business community has used SNA to examine the informal social networks that emerge among members of agencies and ultimately improve the effectiveness and productivity of agencies (Mehra et al., 2006; Morton et al., 2007). We took this approach with the aim of increasing our understanding of interagency collaboration within the local IPV service network for families with young children to inform future improvement efforts.

## Methods

### Study Sample

We identified community-based agencies providing social services to families at risk for IPV in Philadelphia, Pennsylvania. A Research Advisory Committee of 6 members was identified with representation from 6 agencies, including local government human services, maternal and child home visiting, and IPV advocacy organizations. The Research Advisory members provided consultation on the sampling frame. Home visiting agencies and IPV agencies were purposively sampled to saturation (i.e., sampling frame included full representation agencies in these two sectors in the region). Fifty-six agencies were identified and we conducted individual outreach to each of them. Five agencies did not respond after several follow-up attempts; we were unable to find contacts for 3 agencies; 1 agency declined because they do not provide direct service; and the responses from 4 agencies were incomplete or invalid. As a result, 43 agencies were included in the final study sample. This study was deemed by the Institutional Review Board of the Children’s Hospital of Philadelphia as exempt and classified as not-human-subjects research.

### Data Collection

We identified one contact at the director-level at each agency to either participate in the survey or to request participation from staff with knowledge of interagency collaboration. We accepted multiple survey respondents per agency who may have served in a variety of roles in the agency, including administrative, managerial, direct service or clinical service, or other roles.

We developed a social network data collection tool derived from the framework developed by previous SNA on public health systems (Luke & Harris, 2007; Valente et al., 2008). We adapted the framework to fit the context of a system serving families experiencing IPV in Philadelphia. An initial version of the survey was sent to two IPV-agency stakeholders for content review and feedback. Following incorporation of feedback, the survey was pilot tested by two IPV agency respondents (one who was involved in initial survey development and review) for length and clarity. In the final survey, respondents were first prompted to select from the full list of community agencies to identify those with which their own agency has any relationship. For each partner agency selected, the respondent then answered a series of 10 questions that captured 4 types of relationships (see Table 1) about the nature of their partnership with that agency.

**Table 1:** Measurement and Density of Networks

Network	Survey Questions (asked about each partner organization)	# of connections (n)	Density (= # of actual connections / # of *possible* connections = n/903*)
Formal relationship	Does your organization have any shared administration with [Organization X]? (e.g., a shared financial agreement, shared leadership, a board relationship, etc.) Does your organization have a data sharing agreement with [Organization X]?	51	6%
Referral relationship	Has your organization referred a client to [Organization X] for services since March 2020? Has your organization received a referral for a client/patient from [Organization X] for services since March 2020? Has your organization provided any information about a client/patient referred from [Organization X] since March 2020? Has your organization received any information about a client/patient referred to [Organization X] since March 2020?	272	30%
Consultative relationship	Has your organization provided any consultation or coordination for a client/patient case with [Organization X] since March 2020? Has your organization received any consultation or coordination for a client/patient case with [Organization X] since March 2020?	115	13%
Shared activities	Does your organization engage in any shared activities with [Organization X] that are about topics common to both of your agencies? (e.g., city-wide trainings or participation in community meetings) Does your organization engage in any shared activities with [Organization X] that are specific to how your organizations plan to work together? (e.g., developing shared workflows for shared clients or joint working groups)	197	22%

^*^Number of possible connections = A*(A − 1)/2, where A is the number of all agencies in the network. Therefore, the number of possible connections = 43*(43 − 1)/2 = 903

Prior to analyses, the agencies were sorted into 13 categories, including: IPV, maternal and child health home visiting, advocacy, legal services, opioid use/substance use (OUD/SUD) services, mental health, housing and homeless services, respite care, service hub, immigrant and refugee services, reproductive/sexual health services, sexual assault and anti-trafficking, and the Special Supplemental Nutrition Program for Women, Infants, and Children (WIC). The Research Advisory Committee consulted on the organizational categories and the membership in each category for each participating agency. “Advocacy” agencies impact a variety of issues that affect the population of women and children, such as safety (IPV and child abuse), gender equity, reproductive justice, and economic security/power (gender wealth gap). Unlike IPV agencies that provide direct services for IPV-experiencing clients, advocacy agencies may make impact through mechanisms, including but not limited to, philanthropy/funding, increasing public awareness, and policy changes. Agencies in the group of “Service Hub” provide a full range of services to clients and connect their clients with critical external resources, such as healthy meals, health screening, education support, financial assistance, employment support and parenting education and skills. These service hubs serve varying target populations, such as parents with children in foster, kinship, or congregate care, or identification with religious and cultural communities.

### Network Data Management

We used SNA to analyze patterns of collaborations (“ties”) among agencies (“nodes”). There were two key data management steps to transform the original survey responses into an adjacency matrix. (1) The first step was to resolve disagreement between multiple participants within the same agency. When multiple participants completed the survey from the same agency, we collapsed their responses by using the maximum of the responses. That is, if any of the participants from Agency A indicated that Agency A had a connection with Agency B, we documented in the agency-level analytic dataset that A reported to be connected B. (2) The next step was to resolve disagreement between pairs of agencies. In the agency-level analytic dataset, there might be disagreement between pairs of agencies on the same collaboration question. In the primary analysis, we used undirected adjacency matrix and assumed the collaboration to be reciprocal. That is, if either agency reported a collaboration, the undirected matrix considered the pair of agencies to be connected. We chose the approaches to collapse participant’ and agencies’ responses, with two considerations: first, some participants may understandably lack knowledge of the full picture of their agency’s external collaborations because of their specific working roles and experience within the agency and therefore might “under-report” certain types of collaborative activities in the survey; second, because the nature of the survey was an independent research instead of a performance evaluation and survey participants have been guaranteed that their answers will be kept anonymous, there is unlikely to be any motivation for participants to “over-report” their external collaboration in the survey.

### Network Measures

We used R Package ‘Igraph’ (https://igraph.org/r/) for network visualization and statistical analyses. For network statistics, we focused on the interconnectedness (“density”) and prominence (“centrality”) of network members. Previous literature has identified density and centrality as potentially the most informative network measures when examining public health systems (Valente et al., 2007). For instance, networks with high density are more interconnected than networks with low density, providing more paths for communication or dissemination of information. In our study, ‘density’ is calculated as the number of actual connections in a network divided by the maximum possible number of connections. Network density ranges from 0 to 1, with 1 indicating the maximum network density. We used the unit ‘degree’ to measure the centrality of network members. Degree was calculated as the number of ties a node has, theoretically ranging from 0 to 42 (the total number of agencies minus 1).

### Network Visualization

In the graphs, each node represents an agency, and each line connecting a pair of nodes represents the agency-level connections. The size of the nodes is proportional to the “degree” of each agency in the network, such that larger nodes represent agencies with more connections while smaller nodes represent those with fewer connections.

### Identification of Communities within a Network

We also detected and presented communities of agencies (large color-shaded areas in the graphs). Communities are subsets of nodes which are more connected among themselves than to the rest of the network (Girvan & Newman, 2002). We used a clustering algorithm that maximized the “modularity” to detect hidden patterns within the network. Modularity is the fraction of the connections that fall within the given communities minus the expected fraction if connections were distributed at random (Newman, 2006). The modularity maximization method detects communities by searching over possible divisions of a network for one or more that have particularly high modularity.

### Sensitivity Analyses

Two sensitivity analyses were conducted to examine the robustness of results under different approaches of data management. The Sensitivity Analysis #1 was conducted among n = 13 agencies which have more than one staffing type of participant completing the survey. In the data management **(**Sect. "Network Data Management"**)** for the primary analysis (step 1), we combined responses from multiple participants within the same agency, regardless of the differences between these individual participants. In Sensitivity Analysis #1, we examined the networks based on responses from participants with different roles in their agency–administrators/managers versus direct service staffs. In addition, our primary analysis (Sect. "Network Data Management" step 2) assumed the collaboration to be reciprocal if ***either*** agency reported a collaboration between the pair. The Sensitivity Analysis #2 used another approach that required consensus (or mutual understanding), only considering two agencies connected when ***both*** reported a connection between them.

## Results

### Characteristics of Participating Individuals and Agencies

A total of 111 respondents provided data from 43 agencies. Forty percent of the respondents were in managerial roles, 10% were in administrative roles, 42% in direct service or clinical roles, 8% in other roles (e.g., parent educator, legal services, clinical supervisor, advocate, training coordinator). On average, respondents had worked in their agency for a median of 4.8 years (range: 0.5–35 years).

### Overall Interconnectedness

**Fig. 1:**
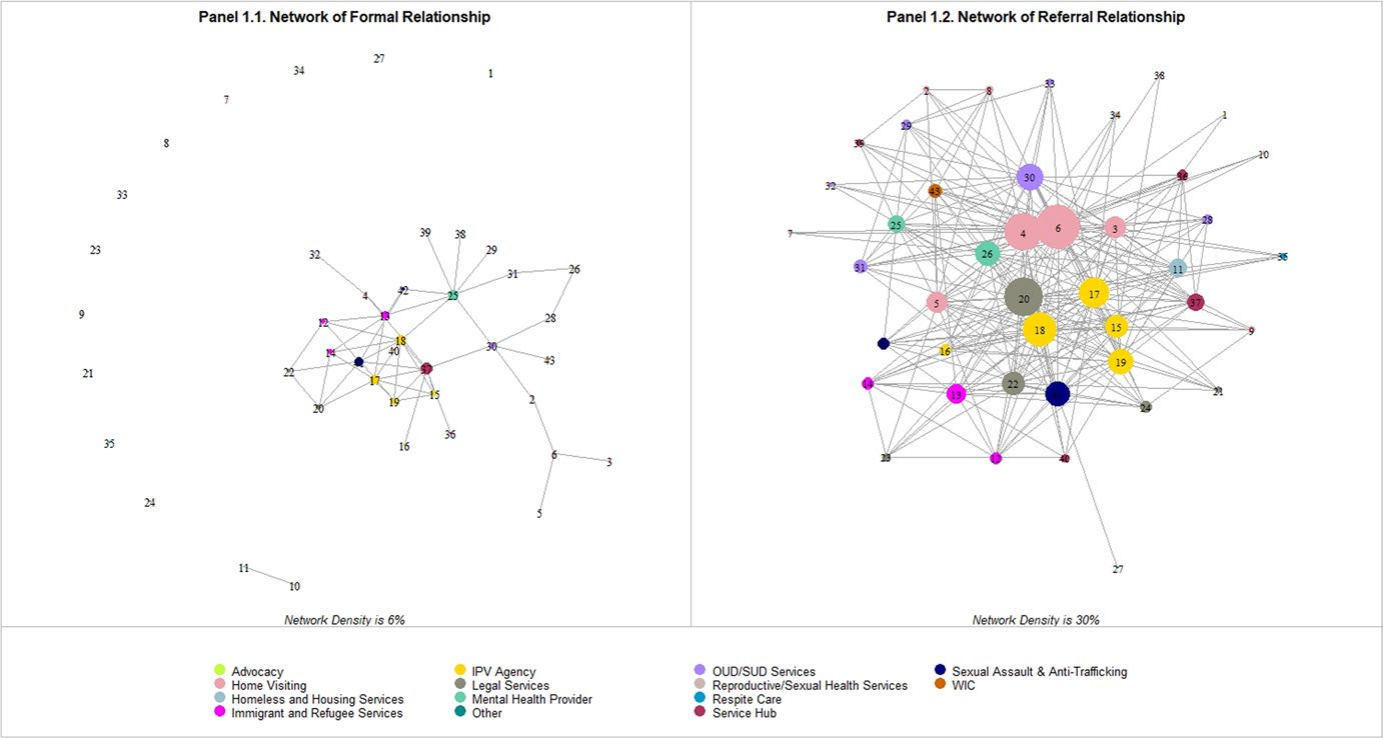
presents the network of formal relationship (Panel 1.1) and the network of referral relationship (Panel 1.2). Each circle represents an agency that responded to the SNA survey question, and each line connecting a pair of circles represents the agency-level connections. The size of the circles is proportional to the “degree” of each agency in the network, such that larger circles represent agencies with more connections while smaller circles represent those with fewer connections. The color of the circles indicates the type of agencies

Table 1 shows the number of connections and density of each network. The network of formal relationship had the lowest number of connections (51) and lowest density (6%), indicating that very few agencies had formal relationships with other agencies, such as shared financial agreement, shared leadership, a board relationship, or data sharing agreement. The network of referral relationship had the highest density (30%), indicating that nearly one third of all the possible referral connections were present. As visual presentation of different density between networks, Fig. 1 presents the maps of the network of formal relationship and the network of referral relationship.

### Centrality of Agencies

We examined the degree (i.e., how many connections an agency has; a measure of centrality) of each agency by network. Table 2 shows the average degree of agencies within each service group. Overall, IPV agencies are more connected than other groups with the highest average degrees in the networks of formal relationship (median = 5), referral relationship (median = 21), consultative relationship (median = 10), and shared activities (median = 18). The median degree of home visiting agencies was lower: on average, a home visiting agency had formal relationship with 1 partner agencies, referral relationships with 7 partner agencies, consultative relationship with 4 partner agencies, and shared activities with 4 partner agencies.

**Table 2:** Centrality (measured by degree) of agencies

Group	# of agencies in the group	Degree of agencies (i.e., # of partner agencies that each agency is connected to) Median (min–max) in the group			
		Formal Relationship	Referral Relationship	Consultative Relationship	Shared Activities
Home Visiting	9	1 (0–3)	7 (3–37)	4 (0–30)	4 (3–38)
OUD/SUD Services	6	2 (0–5)	8 (4–22)	2 (1–5)	5 (2–11)
IPV Agency	5	5 (1–8)	21 (10–29)	10 (5–15)	18 (10–21)
Legal Services	5	0 (0–4)	10 (6–33)	3 (2–11)	13 (4–23)
Service Hub	5	1 (0–9)	7 (2–14)	4 (1–9)	7 (4–8)
Immigrant and Refugee Services	3	4 (4–6)	10 (10–16)	7 (6–8)	12 (8–15)
Mental Health Provider	2	5 (2–8)	17 (14–20)	7 (5–8)	9 (7–11)
Sexual Assault & Anti-Trafficking	2	5 (3–7)	15 (10–20)	5 (3–7)	12 (6–17)
Advocacy	1	0	2	1	7
Homeless and Housing Services	1	1	15	6	5
Other	1	0	1	0	0
Reproductive/Sexual Health Services	1	0	4	3	5
Respite Care	1	0	5	2	2
WIC	1	1	11	3	4

**Fig. 2:**
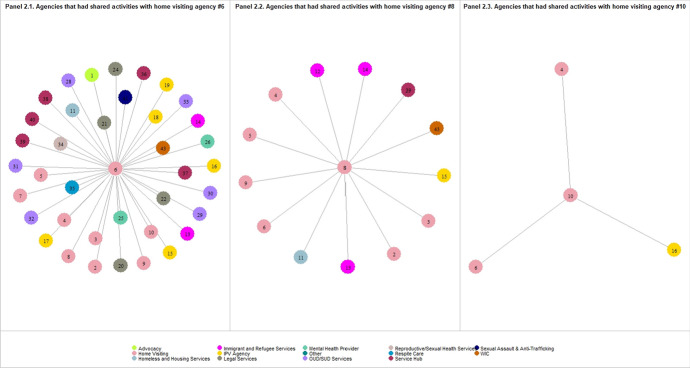
presents the agency-specific networks associated with three home visiting agencies as examples: home visiting agencies #6 (Panel 2.1), #8 (Panel 2.2), and #10 (Panel 2.3). Each circle represents home visiting agencies #2, #8, #10, and their partner agencies. Each line connecting a pair of circles represents the agency-level connections. The color of the circles indicates the type of agencies

Table 2 also shows a wide range (min–max) in the degree of agencies within many groups, indicating a high level of variation in agency connections even within the same service type. The degree of home visiting agencies ranged from 3 to 37 in the network of referral relationship, 0 to 30 in the network of consultative relationship, and 3 to 38 in the network of shared activities. Figure 2 presents the agency-specific networks associated with three home visiting agencies as examples: home visiting agencies #6 (Panel 2.1), #8 (Panel 2.2), and #10 (Panel 2.3) had shared activities with 38, 13, and 4 partner agencies, respectively.

### Communities within Networks

**Fig. 3:**
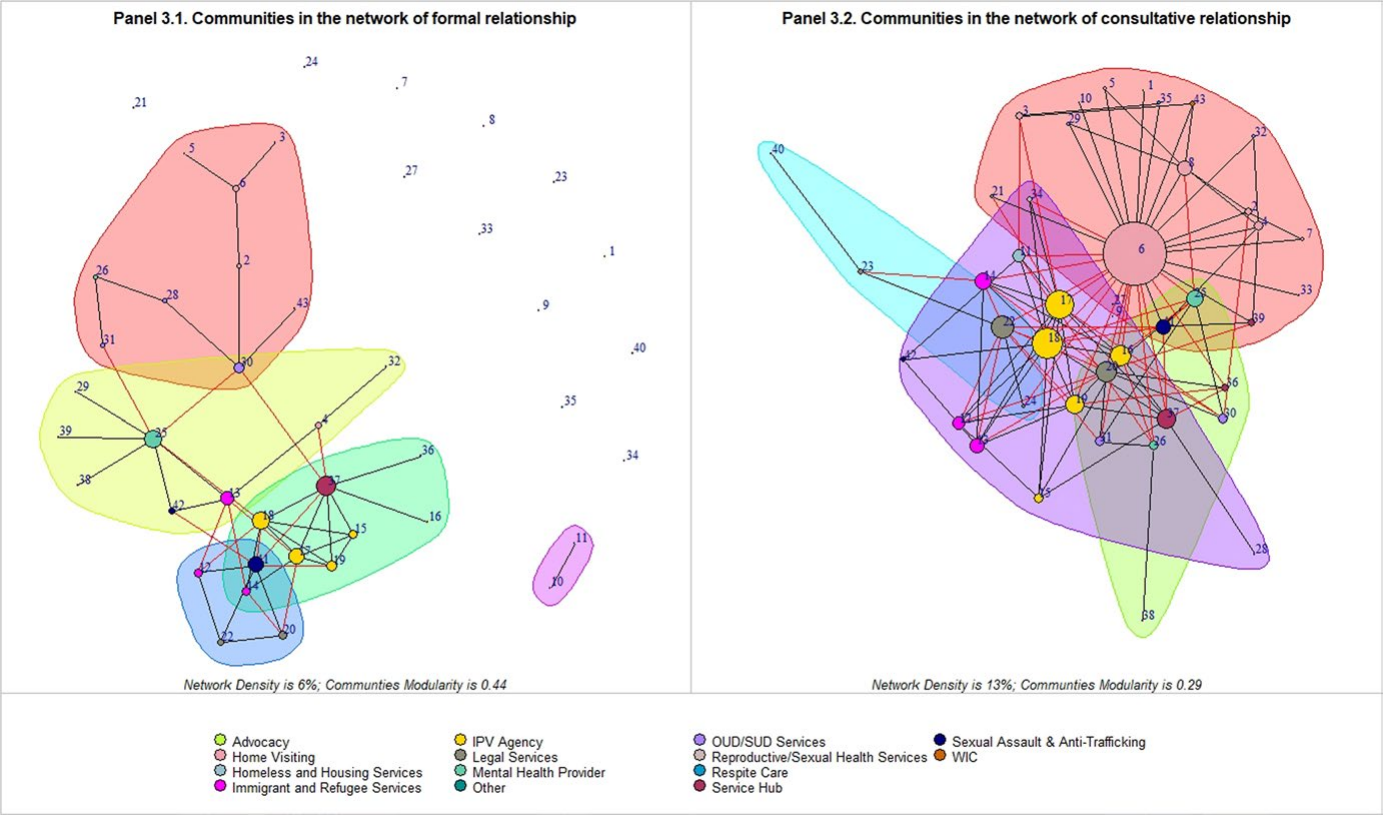
presents the identified communities (the large colored areas in the graphs) within two networks of formal relationship (Panel 3.1) and referral relationship (Panel 3.2). Each circle represents an organization that responded to the SNA survey question, and the color of the circles indicates the type of agencies. The size of the circles is proportional to the “degree” of each agency in the network, such that larger circles represent agencies with more connections while smaller circles represent those with fewer connections. The large colored areas present the identified communities. The black lines indicate connections within communities, and red lines indicate connections between communities

We identified communities, i.e., subsets of agencies which are more connected among themselves than to the rest of the network. Figure 3 presents the identified communities (the large color-shaded areas in the graphs) within two networks of formal relationship and referral relationship. The black lines indicate connections within communities, and red lines indicate connections between communities. Agencies within one community are relatively highly connected with each other (more black connections), while agencies from different communities are relatively loosely connected (fewer red connections). In the network of data sharing agreement (Panel 3.1), five distinct communities were identified: one community (green area) had a high concentration of IPV agencies (#15–19); one community (pink area) had a high concentration of home visiting agencies (#2, #3, #5, and #6) and OUD/SUD agencies (#28, #30, and #31); one community (blue area) had a high concentration of legal services agencies (#20 and #22) and immigrant and refugee services agencies (#12 and #14); and one community (yellow area) comprised a mix of agencies from different sectors; and another community (purple area) only had an IPV agency (#10) and a service hub (#11). The four communities identified in the network of consultative relationship (Panel 3.2) were less distinct: one community (pink area) had a high concentration of home visiting agencies (#2–10); one community (purple area) had a high concentration of IPV agencies (#15–19) and immigrant and refugee service agencies (#12–14); and the other two communities (green area and blue area) share many common members with the pink and purple communities.

### Partner Agencies Connected with Home Visiting Agencies

We further examined service type of the partner agencies connected with home visiting agencies. Table 3 presents the service type of agencies that had a referral relationship with home visiting agencies, relative to the size of groups. Home visiting agencies were most likely to have referral relationships with mental health provider agencies (11 connections, accounting for 61% of all possible connections), OUD/SUD services agencies (26 connections, accounting for 48% of all possible connections), and other home visiting agencies (17 connections, accounting for 47% of all possible connections). There were 13 connections between home visiting agencies and IPV agencies, accounting for 29% of possible connections between these two groups.

**Table 3:** Partner agencies with referral relationships to home visiting agencies

Agencies with referral relationships to home visiting agencies (Home visiting agency–Partner Agency)	# of actual connections between two groups (a)	# of *possible* connections between two groups* (b)	a/b*100%
Home Visiting–Mental Health Provider	11	18	61%
Home Visiting–OUD/SUD Services	26	54	48%
Home Visiting–Home Visiting	17	36	47%
Home Visiting–Respite Care	4	9	44%
Home Visiting–WIC	4	9	44%
Home Visiting–Service Hub	16	45	36%
Home Visiting–Legal Services	15	45	33%
Home Visiting–Immigrant and Refugee Services	8	27	30%
Home Visiting–IPV Agency	13	45	29%
Home Visiting–Sexual Assault & Anti-Trafficking	0	18	0%
Home Visiting–Advocacy	0	9	0%
Home Visiting–Homeless and Housing Services	0	9	0%
Home Visiting–Reproductive/Sexual Health Services	0	9	0%

^*^Number of Possible connections between two groups = Number of home visiting agencies * Number of agencies in the other group. For example, Number of possible connections between home visiting group and OUD/SUD services group = 9*6 = 54. Exception: Number of possible connections between two home visiting agencies (i.e., the row of ‘Home Visiting–Home Visiting’) = 9*(9–1)/2 = 36

### Sensitivity Analyses

**Fig. 4:**
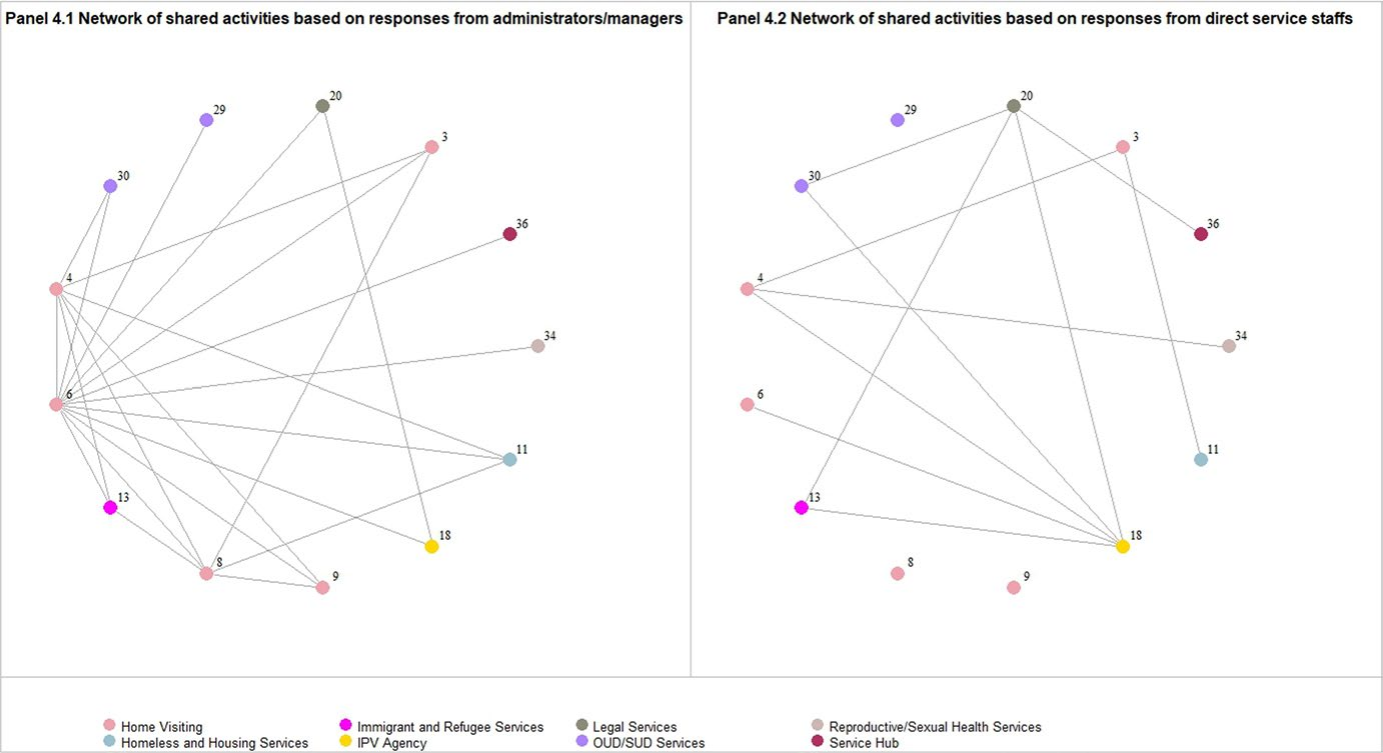
presents the network of shared activities created based on responses from administrators or managers (Panel 4.1) and responses from direct service staffs (Panel 4.2). Each circle represents an organization that has both types of participants completing the survey, and the color of the circles indicates the type of agencies. Each line connecting a pair of circles represents the agency-level connections. The color of the circles indicates the type of agencies

Among the 13 agencies which had both participants with administrative/managerial role and direct service staffs completing the survey (Sensitivity Analysis #1), we found the reported network differed by responses from participants with different roles. Figure 4 presents the maps of the network of shared activities created based on responses from administrators or managers (Panel 4.1) and responses from direct service staffs (Panel 4.2). For most agencies in the analysis, participants with administrative/managerial role reported more connections than their colleagues who are direct service staffs. For a few agencies, such as agencies #18 and #20, the direct services staffs reported more connections than their administrator or manager.

**Fig. 5:**
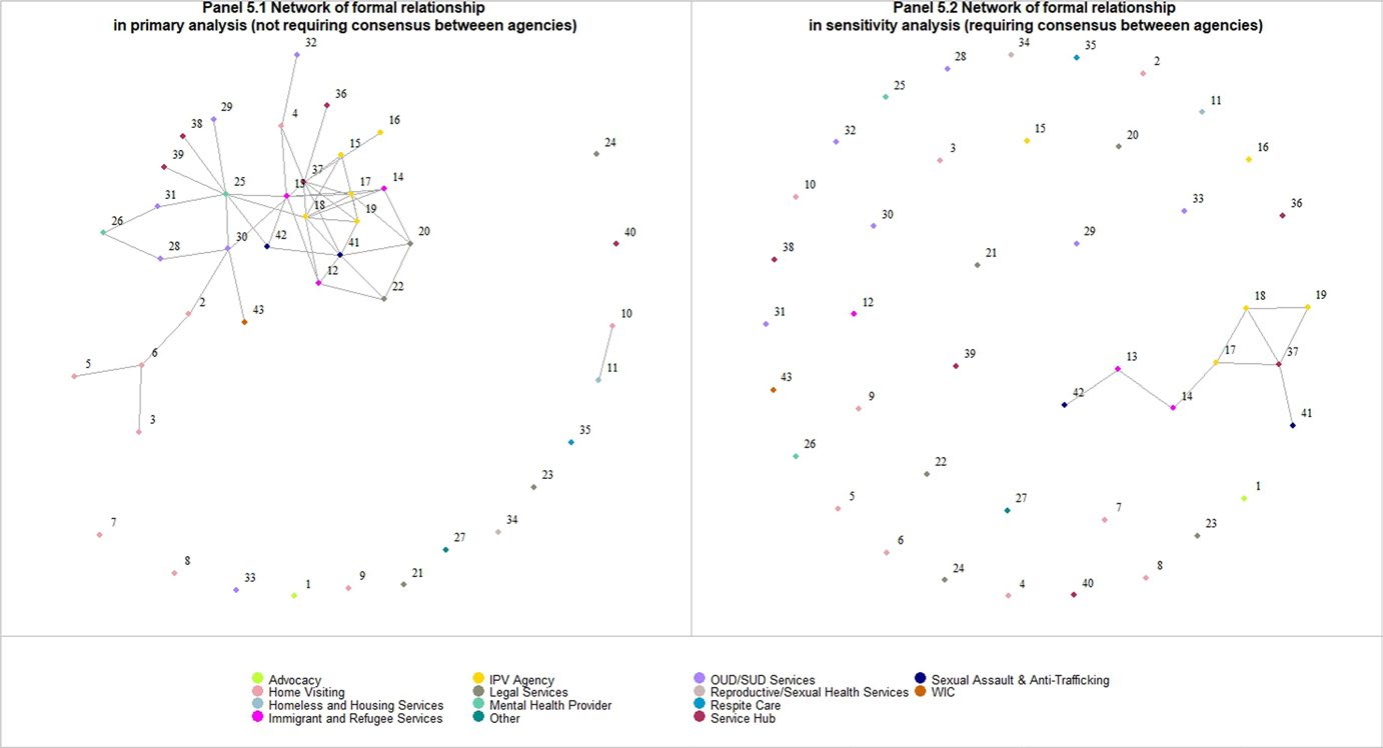
presents the network of formal relationship under two assumptions: Panel 5.1 presents the results when we assumed the collaboration to be reciprocal; Panel 5.2 presents connections between agencies only when a pair had consensus on their relationships. Each circle represents an organization that responded to the SNA survey question, and the color of the circles indicates the type of agencies

In Sensitivity Analysis #2, we illustrated that networks could differ when we required consensus in the reported connections between the pair of agencies. For example, in the network of formal relationship (Fig. 5), Panel 5.1 presents the results using the primary approach (i.e., assuming the collaboration to be reciprocal) while Panel 5.2 presents connections between agencies only when a pair had consensus on their relationships. There were 51 pairs of agencies connected in Panel 5.1 but only 9 connected pairs in Panel 5.2, indicating low level of consensus between agencies regarding the whether they have a formal relationship between them.

## Discussion

### Summary of Findings

This study of the composition and connection of a citywide landscape of community-based agencies serving families at-risk for IPV found a large number of service agencies with low levels of interconnectedness. Making client referrals to each other was the most common form of collaboration between agencies, with infrequent higher-level collaborative relationships such as case consultation or shared administration. IPV agencies were central to overall system networks, having the most connections with other agencies. There was a high level of variation in external collaborations among home visiting agencies, with several home visiting agencies having very few connections in the community but one home visiting program endorsing collaborative relationships with upwards of 38 other agencies in the network. In serving families at-risk for IPV, home visiting agencies were most likely to have referral relationships with mental health provider agencies, OUD/SUD services agencies, and other home visiting agencies. A community detection analysis identified distinct communities within the network and demonstrated that certain agency types were more connected to one another while others were typically siloed within the network. Though IPV agencies were among the most connected agencies identified, IPV and home visiting communities infrequently overlapped.

### Implications for Network Improvement and Service Coordination

Our findings provide a better understanding of the complexity of inter-agency relationships that go into the service system for IPV response and have important implications in facilitating cross-sector collaborative efforts to increase the efficacy of IPV services. We identified an IPV service system for families with young children consisting of diverse sectors within the community; however, the overall inter-agency connectedness was low. Given the complexity of root causes and sequelae of violence for families, a rich service array across health, social, legal and justice sectors are needed to comprehensively support survivors. Low network connectivity indicates the need for considerable work integrating community services to effectively and efficiently serve families. Survivors in an uncoordinated system are often left to seek and navigate multiple services themselves, likely reducing the chances that they will engage in services due to frustration and fatigue with negotiating the myriad set of isolated services (Greeson & Campbell, 2013). Understanding current collaborations, silos, and centrality of agencies within a system is an effective public health tool for identifying areas of focus towards improving ties among agencies. To implement programs for advancing system coordination, the analysis of the centrality of agencies, especially the identification of siloed agencies, can inform effective allocation of scarce resources in a focused way to improve system responses. We also found that individuals serving different roles within the agency may have varying knowledge of their agency’s external collaboration, and for several participating agencies, direct services staffs reported more connections than their administrator or manager. This discrepancy we found emphasizes the importance of universal training and education within agencies to promote knowledge and capacity of IPV-related response and services among all staff. This finding also has an important methodological implication. Some previous organization-level SNA study designs included survey of one primary organization representative, typically at the director level (Costenbader et al., 2018; King et al., 2021; Provan et al., 2004). Our results suggests that, to capture complete SNA data, information should be collected from multiple individuals per agency with varying knowledge of their agency’s external collaborations. In addition, this study identified low levels of consensus between agencies on presence and type of connections, indicating a lack of shared knowledge of community resources. Management structures that promote interagency collaboration should ideally include opportunities for staff to maintain a current awareness of community resources (e.g., encouraged attendance at community resource fairs, coalition participation) and to share this information among peers (Baumgartner et al., 2021; O’Leary et al., 2018; Smith & Mogro-Wilson, 2008). Within the context of saturated service environments, efforts to define best practices for organizations to maintain an up-to-date understanding of community resources are warranted, without clear guidance to date. In future system intervention efforts to promote interagency cohesion, SNA results can be used to allow agencies to visualize and assess their role within the network and to increase their buy-in.

Our study also illustrated high levels of variation in external collaborations among home visiting agencies, with several home visiting agencies having very few connections with other agencies in the community. Previous studies have reported that home visitors often found themselves unable to make appropriate referrals to community resources when necessary, impacting capacity to address IPV (Duggan et al., 2004; Tandon et al., 2005). To build supportive relationships with families and facilitate conversations and care plans responding to violence, home visitors need to be facile and effective in connecting with community-based agencies to better leverage available services and provide families with appropriate referrals and follow-up. Agencies face logistical challenges in care collaborations related to the primacy of confidentiality in addressing the needs of families experiencing IPV. Frameworks such as the Home Visiting Applied Research Collaborative Coordination Toolkit outline the inputs, activities, and outcome metrics, to strengthen inter-agency coordination (The Home Visiting Applied Research Collaborative, https://www.hvresearch.org/service-coordination-toolkit/). Another model in service provision for IPV survivors involves bringing multisectoral case management services together in one location, often referred to as ‘one-stop center’ or ‘one-stop shop’ (Colombini et al., 2008). While the intended results of the one-stop centers model are to increase accessibility, acceptability, quality and coordination of care for IPV survivors, several barriers including insufficient staffing, lack of sustainable funding streams, and inadequate staff trainings, often hinder implementation of this model (Olson et al., 2020). More rigorous evaluation of different models of systems integration is needed to improve holistic support for IPV survivors and their families.

### Limitations

Our study has several limitations. First, we collected data on collaborations among agencies cross-sectionally during the early stage of the COVID-19 pandemic. While we believe our results provide a good general understanding of the systems network in this large city serving IPV-experiencing families, networks are dynamic over time and we were not able to identify how the pandemic impacted the forms and strengths of inter-agency connections. Second, while we accepted multiple representatives serving various roles per agency to participate in the survey, the responding individuals may not be fully aware of their agency’s external collaborations, resulting in under-reporting in the SNA data. Survey participants’ knowledge on their agencies’ external collaborations varied by respondent role. Third, while we focused on formal collaborations reported by agencies, we were not able to measure the quality (e.g., whether a referral was conducted through warm handoff, an evidence-based recommended practice in between-agency referrals), nor the outcome (e.g., whether a IPV survivor has successfully accessed the service of the agency that they were referred to) of the collaborations. Due to heightened confidentiality protections for survivors, often IPV agencies are unable to provide information about the outcomes of services even after a referral has been made unless expressed consent is provided by the client. Fourth, we did not perform a formal associative analysis to further explore the relationship between agency characteristics (e.g., size, model, as predictors) and collaboration level (as outcome) because our current sample size is insufficient for this type of analysis. Future research is warranted to identify the agency-level determinants, barriers, and facilitators for improving the efficiency and quality of interagency connections.

## Conclusion

Our study contributes a first look at the composition and structure of a citywide systems network of community-based agencies serving families at-risk for IPV. SNA methods provide a unique lens from which to provide targeted systems-support efforts. We identified an IPV service system consisting of diverse sectors; however, the overall inter-agency connectedness was low. Our findings suggest opportunities to strengthen maternal and child health systems to provide primary and secondary prevention of IPV among pregnant and parenting women. Understanding current collaborations, silos, and centrality of agencies within a system is an effective public health tool for allocating scarce resources in a focused way to improve system responses efficiently by improving ties among agencies.
